# A coding tool and abuse data for female asylum seekers

**DOI:** 10.1016/j.dib.2020.105912

**Published:** 2020-06-21

**Authors:** Nicole G. Aguirre, Andrew R. Milewski, Joseph Shin, Deborah Ottenheimer

**Affiliations:** a Zucker School of Medicine at Hofstra, Northwell, NY, United States; b Weill Cornell Center for Human Rights, Weill Cornell Medicine, OB/GYN, 1300 York Ave, New York 10065, NY, United States; c Division of General Internal Medicine at Weill Cornell Medicine, New York, NY, USA; d The Nest Community Health Center, Harlem United/Upper Room Aids Ministry, United States

**Keywords:** Asylum, Gender-based violence, Violence against women, Trauma, Abuse, Human Rights

## Abstract

With 1 in 3 women affected, accounting for one billion women worldwide, Violence Against Women (VAW) constitutes one of the widest reaching human rights violations globally. Although the forms they take may vary, these abuses are not confined to a single social class, geographic region, or culture. Existing studies have yet to describe the full burden of abuse that asylum-seeking women endure throughout their lifetimes. We describe a novel coding tool that classifies types of abuse, identifies abuse perpetrators, and estimates how long and how often each abuse was experienced. The authors used this tool to describe and categorize the abuses endured by 85 cisgender, adult women seeking asylum in the United States who presented to the Weill Cornell Center for Human Rights for forensic medical evaluations from 2013 to 2017. We reviewed a total of 180 legal and forensic medical affidavits that were written in support of the applicants’ asylum claims. Using the coding tool, we identified each abuse, classified every perpetrator, and, whenever possible, estimated how long and how frequently each abuse was endured. Interpretations of the raw data contained in this article and a discussion of their significance can be found in our associated publication: “Gender-Based Violence experienced by Women Seeking Asylum in the United State: A Lifetime of Multiple Traumas Inflicted by Multiple Perpetrators” [1]. The coding instrument described herein characterizes VAW by classifying the narrative data that are included in interviews, focus groups, medical records, and the like. Our coding instrument is the first of its kind to describe all types and severities of violence endured by women, classify the perpetrators of that violence, and delineate the timeline of violence over each individual's life. We hope that this holistic approach to classifying and describing VAW will enable other research groups to examine untested or unrealized associations between victims, perpetrators, and abuses. Ultimately, obtaining more complete data will empower us to advocate more effectively and to design more comprehensive care for victims of VAW.

**Specifications Table**

**Table:** 

**Subject**	Forensic medicine
**Specific subject area**	Gender-based violence in female asylum applicants
**Type of data**	Tables
**How data were acquired**	Abuse types, perpetrators, durations, and frequencies were identified in legal and forensic medical affidavits for female asylum applicants and coded in Microsoft Excel spreadsheets.
**Data format**	Raw and filtered
**Parameters for data collection**	Affidvaits were reviewed for cisgender women aged 18 years and older at the time of evaluation, who were applying for asylum in the US and for whom the WCCHR had a legal affidavit and at least one forensic medical affidavit on file.
**Description of data collection**	The authors coded 180 legal and forensic medical affidavits prepared for 85 cisgender, female asylum applicants and employed a novel coding tool to identify and assign numerical codes to every mentioned abuse: The type, severity, perpetrator class, duration, and frequency were coded together with brief, qualitative descriptions for each instance of abuse.
**Data source location**	The legal and forensic medical affidavits are housed at the Weill Cornell Center for Human Rights, a medical student-run organization at Weill Cornell Medicine, New York, NY, USA.
**Data accessibility**	With the article
**Related research article**	Aguirre, N. G., Milewski, A. R., Shin, J., Ottenheimer, D. 2020. Gender-Based Violence experienced by Women Seeking Asylum in the United State: A Lifetime of Multiple Traumas Inflicted by Multiple Perpetrators. Journal of Forensic and Legal Medicine. 72 (2020) 101959. doi: 10.1016/j.jflm.2020.101959

**Value of the Data**

- The data were collected through the application of a novel coding instrument that situates individual instances of abuse in the broader context of an individual's life.
- The data account for multiple types of abuse, various classes of perpetrators, and overlapping timelines of abuse for each individual.
- By capturing detailed descriptions for a comprehensive list of abuses, the data permit a more accurate understanding of the burden of abuse experienced throughout the lifetimes of female asylum applicants than was previously possible.
- The data provide an indicator for the burden of abuse endured by cisgender women seeking asylum in the United States.
- The data-collection strategy suggests a methodology for studying abuse in other populations of women.
- The data can help shape views and policies around the care and protection of women.

## 1 Data description

The data were collected from a set of 180 legal and forensic medical affidavits that were prepared for 85 cisgender women aged 18 years and older who were applying for asylum in the United States and who were evaluated by the Weill Cornell Center for Human Rights (WCCHR) between January 1, 2013 and December 31, 2017. An applicant's affidavits were reviewed only if the WCCHR had both a legal affidavit and at least one forensic medical affidavit on file for the individual. Because the WCCHR offers multiple types of forensic medical affidavits, two forensic medical affidavits were available and reviewed for 10 women, and one forensic medical affidavit was available and reviewed for the remaining 75 women.

The instances of abuse that were reported in an applicant's affidavits were identified and described using the coding tool that is discussed in the Experimental Design, Materials, and Methods section. The supplemental Microsoft Excel file Coded_Abuses_Female_Asylum_Applicants.xlsx contains two spreadsheets. The first spreadsheet, labeled “Applicants by row,” portrays the raw data as they were initially recorded. Each row of the spreadsheet contains the data collected for a single applicant, including: the applicant's age, country of origin, and region of origin; the year that the forensic medical evaluation was performed; the grounds for seeking asylum and the key aspects of the asylum application that the applicant selected from a list of options in an intake form developed by Physicians for Human Rights; and abuse data. Each instance of abuse spans four columns of the spreadsheet wherein the type, perpetrator class, duration, and frequency of the abuse were recorded using numerical labels provided by the coding tool.

The second spreadsheet of the Excel file, entitled “Abuses by row,” displays the data in a different format: Each row corresponds to a single abuse and the spreadsheet contains 853 rows of data. The number of rows assigned to each applicant equals the number of abuses that were recorded for that individual. Rows highlighted in yellow indicate the beginning of the abuse data for each applicant and also contain the applicants’ demographic data. For example, rows 2–9 contain the data for the eight abuses that were recorded for applicant number 1. The next row – row 10 – contains the first abuse recorded for applicant number 2, and so on. Aside from one column that indicates the applicant number and another that displays the abuse number for a given applicant, the columns for this spreadsheet carry the same labels as those in the first spreadsheet.

## 2 Experimental design, materials, and methods

### 2.1 Ethical considerations

The research protocols and coding tool used in this project were approved by the Institutional Review Board of Weill Cornell Medicine, New York, New York, United States [IRB protocol #1,803,019,062].

### 2.2 Creation of the coding tool

Definitions of abuse types and perpetrator classes were synthesized from numerous groups, including the World Health Organization, the U.S. Department of Justice, the United Nations, and the Council of Europe [2], [3], [4], [5], [6], [7], [8], [9], [10], [11], [12], [13], [14], [15], [16], [17], [18], [19]. We devised numerical codes to identify each type of abuse, class of perpetrator, and ranges of duration and frequency. An initial version of the Coding tool was applied to the affidavits for the 14 women who were evaluated by the WCCHR in 2013. The coding tool was iteratively tested and refined on this small subset of data. After finalizing the coding tool, the affidavits from this small pool were reread and re-coded and the finalized coding tool was subsequently applied to all of the remaining affidavits.

### 2.3 The coding tool

#### 2.3.1 Abuse types

Table 1 lists and defines the 14 abuse types and their subtypes. Abuse types 2–5 apply only to adults (age 18 and older) and the versions for minors (under the age of 18) are coded by abuse types 9–12. All other abuse types apply to women and girls of any age. By assigning numerical codes, the tool distinguishes between different forms of abuse and is amenable to statistical analyses.

**Table 1:** Types of abuse.

Code	Term	Definition
1	**FGM/C**	The practice of partially or totally removing the external genitalia of women for non-medical reasons [2].
1a	**FGM/C Type 1**	Partial or total removal of the clitoris and/or the prepuce (the fold of skin surrounding the clitoris).
1b	**FGM/C Type 2**	Partial or total removal of the clitoris and labia minora (the inner folds of the vulva), with or without excision of the labia majora (the outer folds of skin of the vulva).
1c	**FGM/C Type 3**	Narrowing of the vaginal orifice by cutting and bringing together the labia minora and/or the labia majora to create a type of seal, with or without excision of the clitoris.
1d	**FGM/C Type 4**	All other harmful procedures to the female genitalia for non-medical purposes, for example: pricking, piercing, incising, scraping and cauterizating.
1e	**FGM/C Type Unspecified**	FGM/C without a specified type or specific description.
2	**Forced Marriage**	A union wherein at least one person has not given full and free consent to the marriage [3,4].
2a	**Threat of Forced Marriage**	The threat to place or force the applicant into a union wherein she has not freely consented to the marriage.
3	**Sexual Violence**	Any sexual act, attempt to obtain a sexual act, or other act directed against a person's sexuality using coercion, by any person regardless of their relationship to the victim, in any setting [5,6].
3a	**Rape**	Forced sexual intercourse, including vaginal, anal, or oral penetration. Penetration may be by a body part or an object. Rape victims may be forced through threats or physical means. Examples include: Demanding sex regardless of the applicant’s preference. Making her have oral sex against her will. Making her have sexual intercourse against her will. Making her have anal sex against her will. Physically forcing her to have sex. Using an object on her in a sexual way against her will [5].
3a1	**Gang Rape**	Rape that is perpetrated by two or more individuals [7].
3b	**Sexual Assault**	Unwanted visual, verbal, or physical sexual contact [8].
3c	**Sexual Harassment**	Unwelcome sexual advances, requests for sexual favors, and other verbal or physical conduct of a sexual nature that may create an intimidating, hostile, or offensive work or school environment. Submission to or rejection of such conduct might explicitly or implicitly affect an individual’s work or school performance [9].
3d	**Forced Prostitution/Sex Trafficking**	Conditions of control over a person who is coerced by another to engage in sexual activity [10].
4	**Physical Violence**	The application of immediate and unlawful physical force that causes the victim to suffer bodily harm [11].
4a	**Symbolic Violence**	Hitting or kicking a wall, door, or piece of furniture; throwing, smashing, or breaking an object; driving dangerously with the applicant in the vehicle; throwing an object at her [6]; harming someone in her presence in a manner implying that she is also in danger.
4b	**Mild Violence**	Held her down, pinning her in place. Grabbed her suddenly or forcefully. Shook or roughly handled her [6]. Hair pulling, arm twisting, spanking, scratching, or biting [6].
4c	**Minor Violence**	Hair pulling, arm twisting, spanking, scratching, or biting [6].
4d	**Moderate Violence**	Slapping with the palm or the back of the hand; slapping around the face and head [6].
4e	**Serious Violence**	Hitting with an object, punching, kicking, stomping, choking, burning, stabbing, or shooting [6].
4f	**Unspecified Physical Violence**	Physical harm without a specified severity and without a specific description, i.e. “he beat me.”
5	**Psychological/Emotional Abuse**	Any intentional conduct that seriously impairs another person’s psychological integrity through coercion or threats..[AM1] It may affect individuals’ mental health and their social networks, and it may deprive them of opportunities for future personal, social, and economic development. Examples include: isolation from others, verbal aggression, threats, intimidation, control, harassment or stalking, insults, humiliation, and defamation [AM1]I woud delete this sentence. [11].
5a	**Threats of Violence**	Intentional behavior that "would cause a person of ordinary sensibilities" to fear injury or harm, regardless of whether the threatened action was carried to fruition.
5a1	**Threats of Mild Violence**	Shaking a finger or fist, making threatening gestures or faces, or otherwise bullying the applicant [6].
5a2	**Threats of Moderate Violence**	Threatening to harm or damage things the applicant cares about, threatening to destroy her property, or threatening someone she cares about [6].
5a3	**Threats of Serious Violence**	Threatening to hurt or kill the applicant. Threatening her with a knife, gun, club-like object, or other weapon. The perpetrator(s) threaten to commit suicide or act like they want to kill the applicant [6].
5b	**Degradation/Humiliation**	A severe form of emotional abuse that involves the use of intimate information to give private and public insults. It includes such things as “my partner humiliates me in front of others” and “my partner ridicules me” [12].
5c	**Harassment/Stalking**	A course of conduct directed at a specific person that involves repeated—occurring on two or more occasions—visual or physical proximity; nonconsensual communication; verbal, written, or implied threats; or a combination thereof that would cause fear in a reasonable person. As in the model anti-stalking code, the definition of stalking used in the NVAW Survey does not require stalkers to make a credible threat of violence against victims, but it does require victims to feel a high level of fear (“fear of bodily harm”) [13].
5d	**Control/Isolation**	Isolating a person from family and friends; monitoring movements; and restricting access to financial resources, employment, education, or medical care [14].
5e	**Unspecified Psychological Abuse**	Psychological or verbal abuse without a specified severity and without a specific description, i.e. “he was verbally abusive.”
6	**Reproductive Coercion**	
6a	**Forced Pregnancy**	The unlawful confinement of a woman forcibly made pregnant or meddling with contraceptives without the woman's consent in order to make her become pregnant. Forced pregnancy is considered a crime against humanity if it is done "with the intent of affecting ethnic composition of any population or carrying out other grave violations of international law" [11,15].
6b	**Forced Abortion**	The intentional termination of pregnancy without the prior and informed consent of the woman [11].
7	**Kidnapping**	An act of abducting someone and holding them captive [16].
8	**Labor Trafficking**	All work or service that is exacted from any person under the threat of a penalty and for which the person has not offered himself or herself voluntarily [17].
9	**Sexual Violence, Minor**	As defined for adult, but for a woman under the age of 18
10	**Physical Violence, Minor**	As defined for adult, but for a woman under the age of 18
11	**Psychological/Emotional Abuse, Minor**	As defined for adult, but for a woman under the age of 18
12	**Forced Marriage, Minor**	As defined for adult, but for a woman under the age of 18.
13	**Neglect**	Deficit in meeting a person's basic needs, including the failure to provide adequate health care, supervision, clothing, nutrition, housing as well as their physical, emotional, social, educational and safety needs [18].
14	**Honor Killing**	acts of vengeance, usually death, committed by male family members against female family members, who are held to have brought dishonor upon the family [19].

*Shaded boxes indicate types of abuse whose frequencies are coded as “constant/ongoing”.

#### 2.3.2 Perpetrators of abuse

Table 2 lists and defines 8 classes of perpetrators. For abuse types 1,2, and 12, the perpetrator indicates the person who arranged or organized the abuse. Specifically, although a community elder may have performed female genital mutilation/cutting (FGM/C), the perpetrator was coded as the person(s)—i.e. an immediate family member—who arranged to have the applicant undergo FGM/C. Similarly, in the case of actual or threatened forced marriage, the perpetrator was coded as the person(s) responsible for organizing the marriage and not as the prospective husband. For all other abuse types, the perpetrator was coded as the individual(s) who inflicted the abuse on the applicant.

**Table 2:** Abuse perpetrators.

Code	Perpetrator	Definition
1	**Immediate family member**	A parent, sibling, or child of the applicant (i.e. members of the nuclear family).
2	**Extended family member**	Any non-immediate family member of the applicant (examples include grandparents, aunts, uncles, nieces, nephews, cousins).
3	**Intimate partner**	A spouse, boyfriend, or sexual partner of the applicant.
4	**Friend/acquaintance**	A person known to the applicant who is not an intimate partner, immediate family member, or extended family member and who is not identified as belonging to a political group or to a gang.
5	**Political group**	An organization whose members ascribe to the same set of political, social, or cultural ideologies. Such a group may or may not be sanctioned by a government.
6	**Gang member**	A person involved in an organized group of criminals who may or may not be known to the applicant.
7	**Pimp/owner**	A person who arranged for the applicant to take part in prostitution or who trafficked the applicant for sex or labor.
8	**Stranger**	A person unknown to the applicant who is not a pimp/owner and is not identified as belonging to a political group or to a gang.

#### 2.3.3 Categories for duration and frequency

Tables 3 and 4 display the numerical codes for ranges of duration and frequency of abuse. These categories afford graded estimates for quantities that are often vaguely described in affidavits. Codes for an unclear abuse duration and for an unclear abuse frequency were included. Certain types of abuse—degradation/humiliation, neglect, control/isolation, forced marriage, and trafficking—constituted a constant state of existence. These abuses were therefore coded as “constant” in frequency. Conversely, abuses that occurred as one or more punctuated episodes in time, such as beatings, were coded as “discrete” abuses occurring with a specified frequency; the duration of a discrete type of abuse was coded as the timespan from the first to the last episode of the same abuse.

**Table 3:** Duration of abuse.

Code	Definition
1	One-time event
2	One day
3	Up to one week (2–7 days)
4	Up to one month (2–4 weeks)
5	Up to one year (2–12 months)
6	Two to five years
7	Five to ten years
8	More than ten years
9	Unclear from narrative

**Table 4:** Abuse frequency.

Code	Definition
1	Once only
2	Rare: a few times per year
3	Infrequent: a few times per month
4	Weekly
5	Daily
6	Constant/ongoing (applies only to the shaded abuses in Table 1**.**)
7	Unclear from narrative

#### 2.3.4 Definition of a single abuse instance

Repetition of the same abuse (both in type and severity) by the same perpetrator over any timeframe (days to decades) was counted as a single instance of abuse. When a perpetrator simultaneously inflicted multiple abuses of varying severities within the same abuse type, the entire episode was considered as a single instance of abuse and the highest degree of severity was coded. In contradistinction, an abuse type that escalated in severity or evolved into a secondary category (i.e. physical abuse that evolved to rape) at clearly defined intervals was coded as multiple instances of abuse.

### 2.4 Data collection

Under IRB Protocol #1,803,019,062, data were collected from the 180 legal and forensic medical affidavits that were prepared for 85 cisgender women aged 18 years and older who were applying for asylum in the United States and who were evaluated by the Weill Cornell Center for Human Rights (WCCHR) between January 1, 2013 and December 31, 2017. Coding an affidavit entailed identifying every instance of abuse and assigning numerical codes that specified the abuse's type, perpetrator, duration, and frequency. Abuses were pooled and reconciled across all of the affidavits available for an individual applicant. When descriptions of the same abuse differed between affidavits, the more detailed and specific description was adopted. There were a total of four affidavit reviewers: Each affidavit was assessed by at least two members of the research team and the resulting codes were entered into Excel spreadsheets (Version 2013, Microsoft). A third reviewer coded a random subset of affidavits for quality assurance. The entire team reviewed all coder discrepancies and reached a consensus on the final coding for every affidavit.

## Declaration of Competing Interest

The authors declare that they have no known competing financial interests or personal relationships which have, or could be perceived to have, influenced the work reported in this article.

## Appendix Supplementary materials

Image, application 1
